# The Hidden Workload Study protocol: a national mixed-methods analysis of general practice workload and local demographics

**DOI:** 10.3399/BJGPO.2025.0100

**Published:** 2026-04-08

**Authors:** Kirsten Lee, Selma Audi, Thomas Brain, Polly Duncan, Serge Engamba, Tess Harris, Fiona Jones, Jonathan Stewart, Anas Tahir, Jessica Watson, Stephen J Woolford, the Primary care Academic CollaboraTive (PACT)

**Affiliations:** 1 Population Health Research Institute, City St George’s, University of London, London, UK; 2 Centre for Academic Primary Care, University of Bristol, Bristol, UK; 3 Exeter Collaboration for Academic Primary Care, University of Exeter, Exeter, UK; 4 Wellcome-Wolfson Institute for Experimental Medicine, Queen’s University Belfast, Belfast, UK

**Keywords:** general practice, workload, workforce, primary healthcare

## Abstract

**Background:**

General practice workload is increasing. Routinely reported NHS data describes workload in relation to numbers of appointments and clinicians delivering appointments. However, ‘hidden’ aspects of general practice workload, such as administrative and supervisory tasks, are not measured.

**Aim:**

The Hidden Workload Study will examine the full range of tasks that general practice clinicians undertake daily and investigate how workload varies according to clinical role and practice demographics. Participants’ lived experience of workload will also be explored through interviews.

**Design & setting:**

Utilising the Primary care Academic CollaboraTive’s (PACT’s) membership and collaborative methodology, mixed quantitative and qualitative methods will be used. All clinicians working in English general practice, including GPs of all grades, resident doctors, nurses, physician associates, pharmacists, and other allied healthcare professionals will be eligible, aiming for >500 participants across >75 practices.

**Method:**

Participants will collect data on a randomly allocated day in late 2024 or early 2025. Using a data collection form and timers, participants will record their planned work schedule and then all tasks they complete, including all clinical, administrative, and supervisory tasks, and breaks. Practice demographic data will be collected from NHS Fingertips. For the qualitative arm, 15–20 semi-structured qualitative interviews will also be carried out. Quantitative data will be described according to clinical role and practice demographics, and interviews transcribed and reflexively analysed.

**Conclusion:**

The Hidden Workload Study will provide a comprehensive mixed-methods analysis of contemporary general practice workload. Potential explanations for workload variations will be explored, informing future service provision and workforce planning.

## How this fits in

General practice workload is increasing. There is limited literature exploring individual clinicians’ workload in relation to their planned work schedules and their practices’ local demographics. The Hidden Workload Study will examine general practice workload across England using mixed methodology and a real-time data collection approach. This study will provide a deeper understanding of contemporary general practice workload and explore potential explanations for workload variations, informing future service provision and workforce planning.

## Introduction

General practice workload is increasing in England, with associated reports of increasing clinician stress and burnout.^1,2^ Publicly available NHS workload data are limited and primarily measure — and therefore establish targets for — general practice workload according to number of appointments delivered^3^ and the clinical roles providing these appointments.^4^ These data do not account for the ‘hidden’ aspects of workload, such as clinical administrative tasks, practice management, the supervision of colleagues and trainees, unplanned patient contacts, and interprofessional case discussions.^5^ Workload is also affected by patient complexity, which is influenced by a practice’s patient demographics and socioeconomic factors.^6–8^ Workload targets and work schedules may not account for this additional patient complexity, leading to discrepancies between the planned structure of a clinician’s workday and the realities of the work undertaken. Additionally, as numbers of nurses, pharmacists, and other allied health professionals working within general practice grows,^4,9^ it is important to explore how workload might vary among different clinical roles. Finally, routinely reported NHS data do not capture the lived experiences of clinicians and their personal considerations when tackling their daily workload.

Literature is limited on the general practice workload of individual clinicians and the exploration of factors affecting this workload. While previous UK studies have explored general practice workload retrospectively^1,9–12^ or based on clinician recollection,^2,13^ there is little granular data on workload in relation to clinical role or the practice’s patient demographics, such as age, multimorbidity, ethnicity, and socioeconomic status. Additionally, while there are studies that explore workload qualitatively,^14–17^ mixed-methods syntheses with quantitative findings are limited, or have been used to mainly explore other aspects of workload such as operational failures.^18^

### Aims and objectives

The Hidden Workload Study aims to accurately describe all tasks that general practice clinicians undertake on a single day. A mixed quantitative and qualitative methods approach will be used, with objectives divided across two workstreams.

#### Workstream 1 (Workload capture)

- For each participating general practice clinician, to record all work undertaken during an entire workday, including patient contact, administrative, and supervisory work, in real-time, and compare this work to their planned work schedule.
- To describe overall general practice workload in relation to clinical role and key demographic variables of participating practices.
- To describe what proportion of this workload is not captured by routinely collected and publicly reported NHS data.

#### Workstream 2 (Interviews)

- To explore general practice clinicians’ lived experiences of workload.
- To explore general practice clinicians’ opinions about how practice demographics impact their workload.

## Method

### Primary care Academic CollaboraTive (PACT) membership and methodology

Primary care Academic CollaboraTive (PACT) (https://www.gppact.org/) is a UK-wide research collaborative of more than 1000 general practice clinicians, including doctors, nurses, and other allied health professionals,^19–21^ and will be utilised in this study (see Figure 1). Members will collect research data in their own practices, then the data will be combined nationally to increase the power and generalisability of the results. After data collection, PACT members will receive a bespoke practice report, containing anonymised workload data benchmarked against similar practices, and prompts for local quality improvement projects. All participants are required to become a PACT member for at least the duration of the study.

**Figure 1.**
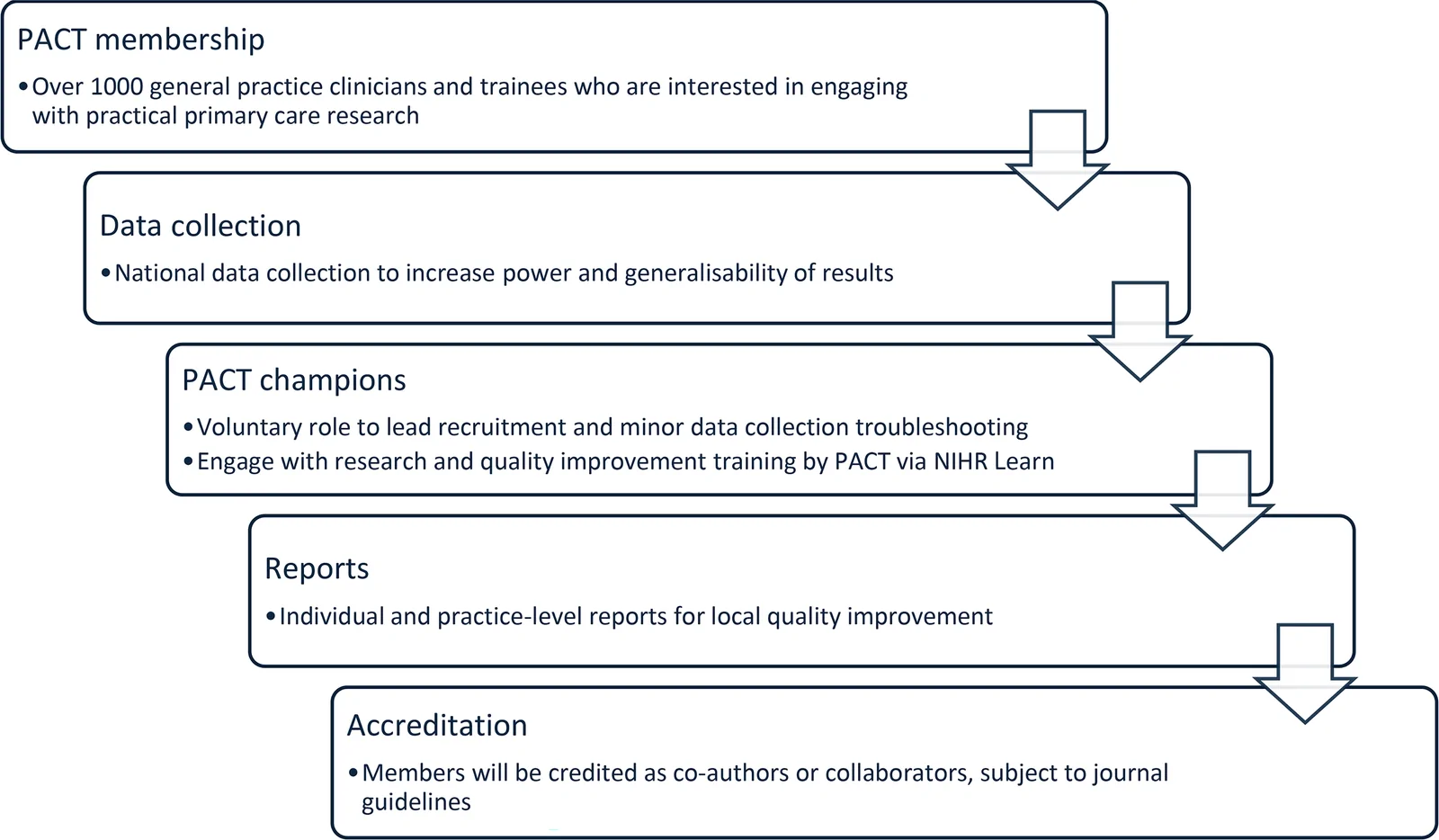
The role of the Primary care Academic CollaboraTive (PACT) network. NIHR = National Institute for Health and Care Research

### Recruitment

The study will recruit general practice clinicians from the PACT membership via email. Non-PACT members will also be recruited via NIHR Research Delivery Network advertising, social media, and communications from relevant professional organisations. Inclusion and exclusion criteria are detailed in Table 1. All participants will be emailed a participant information sheet and an online consent form via REDCap.^22,23^ Participants must inform a practice manager or GP partner of their participation before collecting data. Further purposive recruitment may be undertaken to ensure a range of clinical roles and demographics are recruited. Workstream 1 (Workload capture) aims to recruit at least 500 participants across at least 75 practices.

**Table 1.** Recruitment inclusion and exclusion criteria

Inclusion criteria	Exclusion criteria
Any role involving routine clinical work in a practice setting in England, including, but not limited to: Foundation Year 1 or 2 doctors GP associates-in-training of any grade (ST1, ST2, ST ≥3) GPs (salaried, long-term locum, or partner) Nurses (in any role) Physician associates Pharmacists Paramedics Physiotherapists Healthcare assistants In full-time or part-time employment. Consenting to participate in the study and collect data in the practice where they work.	Working outside of England. Not working on a routine and regular basis in their practice (that is, working on an ad-hoc locum basis, or working <2 sessions a week in their practice). Medical students or other students on a clinical placement in general practice.

Data will be analysed using simple descriptive statistics (see ‘Data analysis’). Although a formal sample size power calculation is possible, this was not performed as there is no single estimate to base it on at this stage.

As part of the consent process, participants can agree to be approached for online interviews as part of Workstream 2 (Interviews); 15–20 interviews are anticipated to achieve adequate power.^24^ Participants will be purposively recruited to achieve a diversity of practice demographics, clinical roles, and experience levels.

### Data collection

Table 2 shows a summary of data that will be collected during the study. Participants will provide their personal demographic details during recruitment, including sex, age, clinical role, time spent working in general practice, practice name, and postcode. Participants’ practice demographic data will then be collected by the research team from NHS Fingertips,^25^ including the number of registered patients, age distribution, deprivation levels, ethnicity, percentage of patients with a long-standing health condition, and life expectancy.

**Table 2.** Summary of data collected

Routinely collected data by participants	
Personal demographics	Sex Age Clinical role Time spent working in general practice Practice name and location
Planned work schedule	Planned contacts Clinical administration Dedicated breaks Supervising Being supervised Meetings and management Personal development
Actual work schedule	Planned contacts (triage, face to face, telephone or video, home visit) Unplanned contacts (face to face, telephone or video, home visit) Clinical administration Dedicated breaks Supervising Being supervised Meetings and management Personal development
Additional tasks	Results (bloods, scans) reviewed or actioned Referral letters Reviewing clinical documents Repeat prescriptions EMIS or SystmOne tasks Emails reviewed or written Texts

Before recruitment, pilot data collection will take place among 5–10 clinicians of varying roles from the research team and PACT committee. Once recruitment has started, further pilot data collection will be done by 5–10 participants outside of the research team or PACT committee. Iterative changes will then be made to the training materials and workload data collection tool based on feedback.

Workload data will be collected between late 2024 and early 2025 to avoid increased winter clinical pressures in December and January. Participants will be randomly allocated a data collection day to achieve even data distribution across the work week. If the allocated data collection day falls on a non-working day (for example, annual leave, study leave, extraordinary meetings and so on), participants may postpone data collection to the next suitable workday, provided they state the reason.

Participants must complete online training on using our data collection tool. Participants will record their planned workday according to their clinic timetable. On their allocated day, participants then record their actual workload according to numbers of patient contacts, time spent on different categories of work using a smartphone timer application, and their clinical administrative tasks (summary of variables can be found in Supplementary data). Participants then input their data online via REDCap. No patient data will be collected and workload data will be pseudo-anonymised before being shared with the participant’s practice.

Semi-structured interviews will be conducted remotely, recorded, and automatically transcribed via Microsoft Teams throughout the data collection window. Interviews are expected to last 30–45 minutes. Interview topics will explore participants’ personal experiences of general practice workload and in relation to their local community. Interview topics will be based on the current literature and the research team’s clinical experiences (see Supplementary data for example interview topics).

### Data analysis

For data validation, a random selection of 5% of paper data collection forms will be reviewed independently by the research team and compared with the digital form completed by the participant. Significant discrepancies will be checked directly with the participant or, if there are trends across multiple participants, will be systematically re-evaluated across the cohort.

Data will be cleaned and categorised by workload category, with actual numbers and time spent on different tasks compared with participants’ planned work schedules. Data will be further stratified and described according to clinical role and practice demographics. Data will then be narratively compared with publicly available NHS workload data, with the workload data that is not routinely reported being described. Stata (version 18.0) will be used to facilitate simple descriptive statistics.

Interview data will be analysed using reflexive methodology. During the data collection window, interviewers will meet regularly and discuss preliminary findings. If certain topics are commonly brought up by interviewees, the interview guide may be changed to explore these topics in more detail. Interviews will be transcribed, cleaned, and anonymised. NVivo (version 1.7.1) will be used to facilitate axial and selective coding to understand common themes in clinicians’ lived experiences of workload and their opinions on how local demographics affect their workload.

Data from both workstreams will be analysed separately and then given equal weighting, using convergent mixed methods to merge and interpret findings.

### Patient and public involvement and engagement (PPIE)

During initial study planning, a draft summary of the study was reviewed by three lay people from the City St George’s, University of London, Research Development Lay Panel and five general practice clinician colleagues of the research team. Feedback was incorporated into the study design and materials. A further patient and public involvement and engagement (PPIE) group of up to five patients and general practice clinicians from across England will be recruited, meeting remotely at least 6 monthly for the study duration. The PPIE group will provide input on study planning, recruitment challenges, data interpretation, and dissemination.

## Discussion

### Summary

The Hidden Workload Study will examine contemporary general practice workload across England using mixed methodology and real-time data collection. This study will provide a deeper understanding of the realities of general practice workload and how different clinical roles and socioeconomic environments may affect workload. Study findings can then be used for future service provision and workforce planning.

### Strengths and limitations

This study will provide English general practice workload data at a level of granularity not found in the current literature, including comparison of planned and actual workload. Furthermore, by synthesising quantitative and qualitative findings, a holistic representation of workload will be generated that is not otherwise possible with routine data. This study expands recruitment beyond doctors, to nurses and other allied health professionals, enabling a direct comparison of workload across different clinical roles and experience levels. Additionally, this study describes workload in relation to practice demographics, adding to existing evidence on the relationship between workload and patient medical and socioeconomic characteristics. Finally, PACT members who participate in this study will receive their own benchmarked research data via a PACT practice report. This can be used to facilitate local quality improvement projects, shortening conventional research delivery timelines.

Limitations of this study include potential selection biases, both from clinicians who may be keener to report their workload difficulties, and from clinicians who have a more manageable workload and feel able to collect data more readily. Furthermore, a key element of this study is contemporaneous data collection. However, self-reporting may lead to inaccurate data recording and time estimates. Other surveys, including the GP Worklife Survey^2^ and the Royal College of General Practitioners (RCGP) Tracking Survey,^13^ have collected workload data retrospectively. However, this may instead be subject to recall bias and other inaccuracies. While an embedded researcher or an ethnographic approach might address these challenges, as used in other workload studies,^17,18^ this study has been designed to collect data in a broad and naturalistic way, maximising national recruitment across as many roles and experience levels to increase the generalisability of results.

### Implications for research and practice

The Hidden Workload Study aims to address the current limitations of general practice workload data, both within academic literature and NHS data. Study findings will focus on comprehensive workload reporting, clinical role comparisons, patient demographic relationships, and qualitative insights. Practice reports could allow participants to identify aspects of their workload that differ from others in similar roles. This could help facilitate local quality improvement; for example, adoption of workload efficiency strategies. More broadly, results may assist policymakers in better understanding the full scope of work undertaken by general practice clinicians, and the potential contributors to this workload. The inclusion of the clinician’s lived experience within the study’s findings may also inform strategies to prevent burnout and improve job satisfaction, contributing to staff recruitment and retention. Data can better inform interventions from government and NHS leadership, creating more appropriate changes to workforce planning and service provision.
